# The Potential Role of miRNA-Regulated Autophagy in Alzheimer’s Disease

**DOI:** 10.3390/ijms23147789

**Published:** 2022-07-14

**Authors:** Hu Zhang, Jiling Liang, Ning Chen

**Affiliations:** 1 Graduate School, Wuhan Sports University, Wuhan 430079, China; 17858503393@163.com (H.Z.); ljl19930210@163.com (J.L.); 2 Tianjiu Research and Development Center for Exercise Nutrition and Foods, Hubei Key Laboratory of Exercise Training and Monitoring, College of Health Science, Wuhan Sports University, Wuhan 430079, China

**Keywords:** Alzheimer’s disease, miRNA, autophagy, exercise, pharmacological therapy, natural products

## Abstract

As a neurodegenerative disease, Alzheimer’s disease (AD) shows a higher incidence during the aging process, mainly revealing the characteristics of a significant decrease in cognition, uncontrolled emotion, and reduced learning and memory capacity, even leading to death. In the prevention and treatment of AD, some pharmacological therapy has been applied in clinical practice. Unfortunately, there are still limited effective treatments for AD due to the absence of clear and defined targets. Currently, it is recognized that the leading causes of AD include amyloid-β peptide (Aβ) deposition, hyperphosphorylation of tau protein, neurofibrillary tangles, mitochondrial dysfunction, and inflammation. With in-depth mechanistic exploration, it has been found that these causes are highly correlated with the dysfunctional status of autophagy. Numerous experimental results have also confirmed that the development and progression of AD is accompanied by an abnormal functional status of autophagy; therefore, regulating the functional status of autophagy has become one of the important strategies for alleviating or arresting the progression of AD. With the increasing attention given to microRNAs (miRNAs), more and more studies have found that a series of miRNAs are involved in the development and progression of AD through the indirect regulation of autophagy. Therefore, regulating autophagy through targeting these miRNAs may be an essential breakthrough for the prevention and treatment of AD. This article summarizes the regulation of miRNAs in autophagy, with the aim of providing a new theoretical reference point for the prevention and treatment of AD through the indirect regulation of miRNA-mediated autophagy.

## 1. Introduction

Alzheimer’s disease (AD) is a high-incidence neurological disease in the elderly. With the advent of global aging, AD patients show an increasing trend, with an estimated number of more than 113 million in 2050 [1]. In 2020, the average annual cost of AD treatment was approximately 1.09% of gross domestic product (GDP) in most countries [2]. Therefore, the high incidence of AD aggravates the pressure on social medical resources and family finances. The prevention and treatment of AD have become a significant challenge for clinical and basic medicine. AD mainly includes early-onset family AD and late-onset AD. There are different theoretical hypotheses for the primary pathogenesis, including the amyloid cascade hypothesis, the tau protein hypothesis, the ischemia etiology hypothesis, the cholinergic hypothesis, and excitotoxicity [3,4,5,6]. For clinical treatments of AD, a series of drug developments have also been carried out based on the above hypothesis, mainly including the development of cholinesterase inhibitors and immunotherapy, monoclonal antibodies, and amyloid-β peptide (Aβ) and tau protein [7] -targeted therapeutic drugs. However, some drugs currently used for clinical intervention have toxic or side effects, which are not conducive to long-term application in elderly patients. Therefore, it is imperative to seek novel and effective methods for the prevention and treatment of AD. With an in-depth understanding of autophagy in diseases, it has been found that the abnormal functional status of autophagy may be an important factor leading to AD, and regulating the functional status of autophagy has also become one of the effective ways to intervene and treat AD at the early stage [8]. At present, relevant basic studies have confirmed the regulation of autophagy through exercise, fasting, metformin, and resveratrol [9,10,11].

In addition to the above regulatory pathways, some microRNAs (miRNAs) also show abnormal changes in neurodegenerative diseases and may be involved in the regulation of autophagy in disease states [12]. Therefore, targeting miRNAs to regulate the functional status of autophagy may be an important research direction for the prevention and treatment of AD in the future. It is also a potential strategy to achieve clinical treatment efficacy. In order to further understand the relationship between miRNAs and autophagy in AD, this article conducts a systematic classification, and summarizes the miRNAs involved in the regulation of autophagy, thus hoping to provide a relevant theoretical reference point for targeting miRNAs to prevent and treat AD through regulating the functional status of autophagy in the future.

## 2. Autophagy-Related Molecular Mechanisms

Autophagy, as the core mechanism of intracellular material recycling, participates in cellular cycling by decomposing aging or damaged organelles and mis-folded proteins into basic substances such as amino acids. Autophagy mainly includes macroautophagy, microautophagy, and chaperone-mediated autophagy [13]. Macroautophagy mainly wraps organelles and macromolecular substances in double membrane-forming structures, with the combination of lysosomes, to realize the degradation process. Chaperone-mediated autophagy is the degradation of substrates mediated by specific molecular chaperones into lysosomes, while microautophagy is the process of degrading substrates through invagination of lysosomal membranes. Although there are differences between the three processes, all of them finally involve lysosomes, so autophagy is also named a lysosome-dependent degradation pathway [14]. However, the autophagy mentioned so far is mainly macroautophagy (hereafter referred to simply as autophagy). Specific conditions such as starvation, exercise, and hypoxia are important means to stimulate the induction of autophagy. Its basic molecular mechanisms include the involvement of adenosine 5′-monophosphate (AMP)-activated protein kinase (AMPK) and mammalian target of rapamycin (mTOR) to induce and inhibit autophagy, respectively.

**Figure 1:**
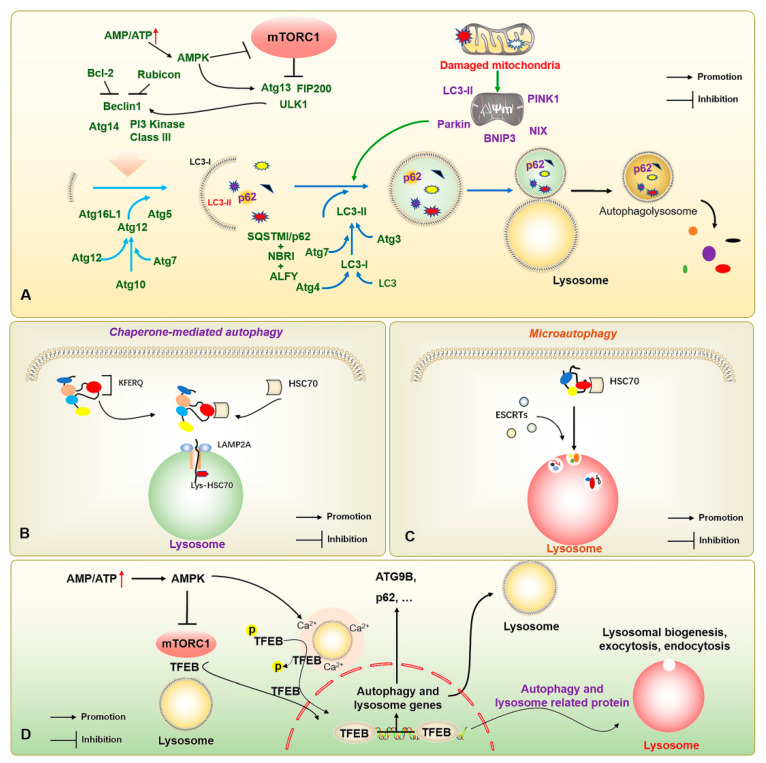
The molecular mechanisms of macroautophagy, chaperone-mediated autophagy, microautophagy, and autophagy-lysosome-associated transcription factors. (**A**) The increased AMP/ATP ratio activates AMPK to inhibit mTOR; and activates the ULK1 complex (ULK1-ATG13-ATG101-FIP200) to further induce the completion of autophagy process through a series of autophagy-related proteins, including PI3K-ATG2-ATG18 complex, and ATG5-ATG12-ATG16L1 complex. (**B**) HSC70 realizes the degradation by recognizing the KFERQ motif and transporting the substrate protein into lysosomes through LAMP2A. (**C**) HSC70 binds to KFERQ-carrying proteins and degrades the substrates through ESCRT complexes for accomplishing substrate transport and lysosome phagocytosis. (**D**) The increased AMP/ATP ratio activates AMPK for inhibiting mTOR and regulating the lysosomal Ca^2+^ microenvironment to promote the nucleus translocation of TFEB after dephosphorylation, thereby binding autophagy- and lysosome-related genes, and enhancing autophagic flux. The ↑ in red color represents the increased AMP/ATP ratio; the → and ┤ in black color represent the promotion and inhibition, respectively.

The autophagic process in mammalian cells is finely regulated by a series of autophagy-related genes (ATGs), mainly through the activation of class III phosphatidylinositol 3-kinase (PIK3C3) after autophagy is triggered by the UNC-51-like kinase 1 (ULK1) complex (ULK1-ATG13-ATG101-FIP200). The complexes (BECN1, AMBRA1, ATG14L, VPS15, and VPS34) recruit relevant proteins, including the PI3K-ATG2-ATG18 complex, and complement and facilitate lipid transport through the transmembrane protein ATG9, as well as recruit the ATG5-ATG12-ATG16L1 complex to promote autophagic membrane elongation and formation. Finally, the modification of phosphatidylethanolamine (PE) is completed by ATG8-family proteins to complete the induction of autophagy, and the degradation of substrates is achieved by binding to lysosomes. The microtubule-associated protein light chain 3 (LC3) at a cytosolic form (LC3-I), a member of the ATG8 family mainly composed of LC3/GABARAP, binds to PE to form an LC3-phosphatidylethanolamine conjugate (LC3-II); it thereby attaches to the inner membrane of autophagosomes for promoting and extending autophagic vesicles to encapsulate the products to be degraded and the autophagic substrate protein Sequestosome 1 (SQSTM1/p62), finally degrading the substrate upon binding to lysosomes [15,16]. However, some organelles have unique styles of autophagy, such as mitophagy. Mitochondria are the primary source of energy in the body. The occurrence of mitophagy is mainly responsible for the timely removal of aging and damaged mitochondria ensuring the quality of mitochondria in cells to maintain the regular operation of the body. Molecular pathways such as the signal pathways with the involvement of PTEN-induced putative kinase protein 1 (PINK1)/Parkin, BCL2/adenovirus E1B 19kDa-interacting protein 3/Nip3-like protein X (Bnip3/Nix), and FUN14 domain containing 1 (FUNDC1) are mainly responsible for regulating mitophagy. In the PINK1/Parkin signal pathway, PINK1 protein enters the mitochondrial space by binding to the mitochondrial outer membrane, but it is rapidly degraded when it contacts the mitochondrial inner membrane and loses its functional activity, so the content of the PINK1 in the standard mitochondrial matrix is low. In mitochondria, PINK1 directly binds to the outer membrane and induces the E3 ubiquitin ligase Parkin to participate in the initiation and progression of mitochondria-specific autophagy. Between Bnip3 and Nix, Nix promotes mitophagy by dissociating Bcl-2/Beclin1 binding, thereby recruiting Parkin to the mitochondrial outer membrane, and participating in the recruitment of LC3 to mitochondria. FUNDC1, a mitophagy-related protein, can be activated under hypoxic conditions and induce mitophagy by interacting with LC3-II and participating in the corresponding regulation of mitochondrial dynamics (Figure 1A) [17].

In addition, there are molecular chaperone-mediated autophagy and microautophagy. Of these, molecular chaperone-mediated autophagy does not depend on the vesicle delivery of metabolic wastes, but mainly on the specific recognition of pentapeptide motif (KFERQ) target proteins and receptor proteins on the lysosomal membrane by the chaperone protein heat shock cognate 70 kDa protein (HSC70) [18]. When the target protein binds to the lysosome-associated membrane protein 2A (LAMP2A) and enters the lysosome, Lys-HSC70 is responsible for mediating the transport of the degradation substrate in the lysosome to finally be degraded into basic components for recycling under the action of hydrolase (Figure 1B). During endosomal microautophagy, HSC70 binds to proteins carrying a KFERQ protein or KFERQ-like motifs and relies on endosomal sorting complexes required for transport (ESCRT)-I/II/III to achieve substrate transfer (Figure 1C). The heteromers of ESCRT-I and ESCRT-II and the monomers of ESCRT-III can be degraded intracellularly. When ESCRTs are recruited to the lysosomal membrane to form a complex, they induce the invagination of lysosomal membrane to wrap the substrate into the lysosome for degradation. When the lysosomal membrane encapsulates the substrate into the lysosome, ESCRT-III dissociates from the complex for recycling. Notably, autophagy-related transcription factors, such as the transcription factor EB (TFEB), also play important roles in regulating autophagic vesicle formation, lysosomal regeneration, and vesicle-lysosome binding, while transcription factors including the TFEB also play an important role in the regulation of autophagy [19] (Figure 1D). However, with the refinement of research in the field of autophagy, more signal pathways, underlying mechanisms, and regulatory proteins associated with autophagy have been discovered [20], which also offer the possibility for more refined regulation of autophagy.

## 3. The Functional Status of Autophagy in AD

### 3.1. Role of Autophagy Dysregulation in AD

With the aging of the body, the abnormal changes of autophagy in the nervous system may be an important factor for inducing AD. According to current studies, the optimal functional status of autophagy can accelerate the removal of accumulated Aβ, excessive phosphorylated tau protein and neurofibrillary tangles, dysfunctional mitochondria, and harmful small molecular substances in neurons, but the abnormal accumulation of these factors will also reverse to inhibit autophagy or impair autophagic flux [21], thus forming a vicious circle. Current studies mainly show that the level of autophagy in hippocampal tissues of AD subjects is significantly decreased, but some studies have also shown that there may be an increased level of autophagy during the aging process; this may be related to the induced autophagy in a feedback manner caused by the increase in aging-induced impaired organelles and proteins in the body, and the formation of more non-functional autophagic vesicles inside. The up-regulation of autophagy genes has also been confirmed in the hippocampus of AD model mice in the relevant literature, but with the progression of the disease, the clearance rate of substrates gradually decreased, and there was no interaction between autophagic vesicles and lysosomes. The apparent increase in binding suggests that there is an abnormality in autophagic flux, and the up-regulation of autophagy may be a feedback mechanism in the disease process [22]. Previous reports have documented the existence of large numbers of immature autophagic vacuoles in brain tissues of AD patients [23,24] for the first time, suggesting deficient autophagy during the progression of AD. Some studies have also shown that local Ca^2+^ dysfunction in neurons can lead to a decrease in neuronal axonal transport capacity, thus leading to synaptic dystrophy and inflammation, and in turn inducing neurodegenerative diseases, which may be associated with decreased lysosomal acidification [25,26]. In addition, it has also been found that the acidification of lysosomes in neurons is decreased before the deposition of Aβ in the development of AD, and the accumulation of a large number of Aβ-positive autophagic vesicles (Avs) into large perinuclear vesicles may be responsible for the major cause of amyloid plaque [27]. Therefore, the abnormal function of autophagy may promote the occurrence and development of AD at the different stages of the disease or in patients with various brain insults. Exploring the changes in the functional status of autophagy during the development process of AD may be of great significance for the prevention and treatment of AD.

### 3.2. Regulatory Role of Autophagy in AD

As a common aging-related neurodegenerative disease, AD shows brain atrophy during the progression of the disease, especially cortical tissue; however, as the disease symptoms develop, including declined learning and memory capacity, the reduced function of hippocampal neurons may be the major trigger. With the gradual reduction of body functions, the autophagy-lysosomal system in the nervous system shows a low efficiency for the maintenance of cellular homeostasis, which may be one of the essential reasons for the triggering of AD [28]. The functional status of autophagy is also an essential mechanism for controlling the occurrence and progression of AD [29]. In the pathogenesis of AD, the excessive deposition of Aβ and the excessive and abnormal phosphorylation of tau protein are currently recognized inducers and pathological biomarkers. In addition, low mitochondrial quality and high neuroinflammation are also known as important triggers for AD, and an abnormal functional status of autophagy is an essential factor for aggravating these triggers. In healthy adults, the production and clearance rates of Aβ are 7.6% and 8.3% per hour, respectively [30], and the excessive accumulation of Aβ in AD may be closely related to the decreased clearance rate. Subsequently, studies have demonstrated down-regulated Beclin1 in the brain tissues of AD patients. Similarly, knockout Beclin1 can result in abnormal amyloid precursor protein (APP) processing and the increased Aβ accumulation [31]; in contrast, overexpressed Beclin1 in hippocampal tissue can stimulate a significant reduction of accumulated Aβ in brain tissue [32]. Deleting autophagy-related genes such as Atg5 and Rubicon can lead to an excessive deposition of Aβ in mouse neurons, while promoting LC3-associated endocytosis can effectively enhance the clearance of neuronal Aβ and the mitigation of AD symptoms [33]. The APP mutants and Aβ in hippocampal neurons also can trigger the defective mitophagy and mitochondrial fragmentation, thereby resulting in the impairment of neuronal functions [34], while increasing the level of PINK1/Parkin can dynamically optimize the balance between mitochondrial fusion and mitochondrial fission. Increasing mitophagy also effectively promotes the clearance of damaged neuronal mitochondria and accumulated Aβ, thereby realizing the improvement of learning and memory capacity [35,36]. TFEB, as an important regulator of autophagy and lysosome, also presents a significant decrease in AD, and the targeted activation of TFEB can effectively activate autophagy, improve the autophagy-lysosomal pathway, reduce APP, Aβ, tau protein and improve learning and memory ability in mice [37,38]. In addition, increasing the level of Klotho protein can also avoid the neuronal injury to execute the preventive role of AD, which may be related to promoting the formation of ULK1 complex, inhibiting the IGF-1/PI3K/Akt/mTOR signaling pathway, and up-regulating TFEB for inducing autophagy [39]. Similarly, reticulophagy, lipophagy, nucleophagy and other subtypes of autophagy also show abnormal changes during the progression of AD [40]. However, another study has shown that the transcription of autophagy-related genes and corresponding protein expression are up-regulated in AD, which may be due to compensatory regulation [41]. Therefore, the functional status of autophagy is highly correlated with occurrence and progression AD [42]. Moreover, the deficiency of autophagy in the cerebral cortex is also an important factor for leading to the further development and deterioration of AD [43]. The abnormal functional status of autophagy in AD and AD-related incentives can form a vicious circle, so that regulating the functional status of autophagy is likely to become an essential breakthrough in the prevention and treatment of AD, as shown in gradual confirmation through exercise [44], metformin [45], and resveratrol [9] interventions.

## 4. miRNAs Involved in AD

The miRNA is mainly formed by RNA polymerase II in the nucleus. The pri-miRNA is formed by the directed catalytic cleavage of the RNAase III-related proteins Drosha and Pasha to form a pri-miRNA of about 70 nucleotides. Followed by the transportation to the cytoplasm by RNA-GTP and Exportin 5, the pri-miRNA is cleaved by Dicer to produce a double-stranded non-coding RNA approximately 22 nucleotides in length, and finally mature single-stranded miRNA by the RNA-induced silencing complex (RISC). The single-stranded miRNA achieves complete and incomplete binding of the 3′ or 5′ end of the targeted mRNA through complementary base pairing, and inhibits the transcription process of the corresponding mRNA, the translation process of the encoded protein, and then participates in the physiological regulation of cells and organisms. With the in-depth studies of miRNAs, a series of miRNAs have been identified and confirmed to be involved in multiple diseases, and regulating miRNAs has become a potential means for the prediction, diagnosis, prevention, and treatment of diseases [46]. As a degenerative neurological disease, AD is accompanied by changes of many miRNAs during the occurrence and development of the disease, as well as the adaptation of the body [47]. During the pathogenesis of AD, excessive Aβ deposition, the phosphorylation of tau protein and neurofibrillary tangles, reactive oxygen species (ROS) accumulation, mitochondrial dysfunction, decreased synaptic plasticity, and abnormal neuronal cell cycle, neuronal apoptosis, and abnormal autophagy can be controlled by miRNAs.

In human serum, the up-regulated miR-24-3 and down-regulated miR-193a-3p may promote neuronal apoptosis [48,49]; miR-4422-5p can target gamma-secretase-activating protein (GSAP) and beta-site APP-cleaving enzyme-1 (BACE1) to exacerbate Aβ formation [50,51]; the down-regulated miR-148a-3p can trigger the neurotoxicity of Aβ to the nervous system [52]; the reduced levels of miR-222, miR-223, miR-29c-3p, and miR-19b-3p in blood samples of AD patients, and the increased level of miR-501-3p, have the potential to predict the occurrence and progression of AD [53,54,55,56]. At the same time, miR-331-3p may have a potential neuroprotective effect in AD by inhibiting neuroinflammation [57], the elevated miR-1273g-3p can aggravate mitochondrial damage and Aβ production [58], and miR-545-3p, miR-222, miR-125b, miR-455-3p, and miR-34a-5p have been confirmed to have the potential for the diagnosis and treatment of AD [59]. Another study has reported that up-regulated miR-146a can promote tau protein hyperphosphorylation [60]. In addition, down-regulated miR-146a and miR-181a may herald the transition from mild cognitive impairment to AD [61], and elevated levels of miR-206 in olfactory mucosa can be a biomarker for AD [62] diagnosis (Table 1).

Increasing miRNAs have also been found to be involved in the occurrence and development of AD. In cell and animal models of AD, the down-regulated miR-433 exacerbates Aβ-induced reduction of neuronal viability [63], while the up-regulated miR-206 and miR-613 can inhibit the expression of the brain-derived neurotrophic factor (BDNF) [64,65]. In brain tissues of AD mice, the down-regulated miR-200c can exacerbate tau protein phosphorylation in brain tissues and reduce learning and memory capacity of the mice [66]; the elevated miR-34c can reduce synaptic plasticity [67], the reversal of miR-29c-3p can suppress the targeting of BACE1 to activate the Wnt/β-catenin signaling pathway [68], and the elevation of miR-155 can induce neuroinflammation and cognitive impairment [69,70]. Meanwhile, increased miR-361-3p and miR-340 can target BACE1 to inhibit Aβ accumulation [71,72], while elevated miR-128 can aggravate Aβ production, APP formation, and inflammatory responses [73]. In addition, the up-regulated miR-30b can also exhibit neuronal synapse disruption and reduced cognitive capacity [74], and miR-98 can suppress the Notch-signaling pathway to exacerbate Aβ production and to result in excessive oxidative stress and mitochondrial dysfunction [75] (Table 2). The abnormal changes in miRNAs in blood, cerebrospinal fluid, cortex, and hippocampal tissues can have a significant effect on the occurrence and development of AD. Therefore, miRNAs may become important potential biomarkers for the prediction, diagnosis, prevention, and treatment of AD, thereby providing novel directions for the prevention and treatment of AD. However, due to the multiple targets of miRNAs, the precision and targeted prediction and treatment of AD through targeting miRNAs are still complex, and need to be further explored.

## 5. miRNA-Mediated Autophagy in AD

**Figure 2:**
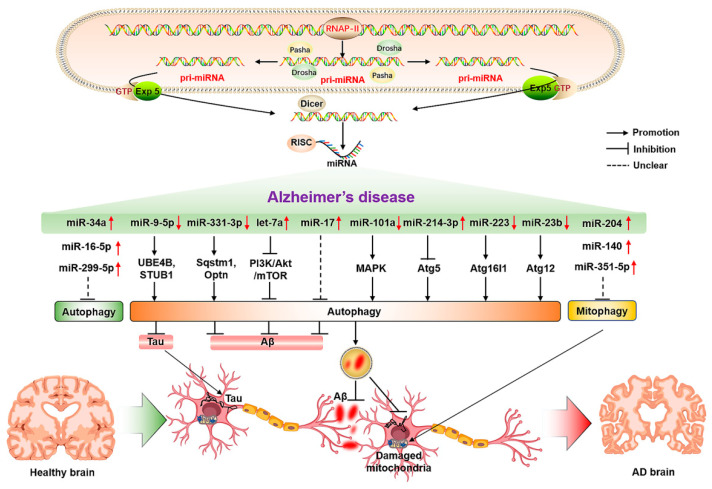
The molecular mechanisms of miRNA-mediated autophagy in AD. The ↑ and ↓ in red color represent the up-regulated and down-regulated microRNAs, respectively. The → and ┤ in black color represent the promotion and inhibition, respectively; the ┈ in black color represents the unclear promotion or inhibition.

As a highly conserved material recycling system in eukaryotic cells, autophagy requires the precise coordination of many autophagy-related proteins. Therefore, miRNAs are also involved in regulating autophagy-related genes in the process of regulating protein translation, and abnormal changes in miRNAs that can regulate autophagy-related proteins in the process of AD may be an important factor for triggering and aggravating AD. Relevant studies have shown that the inhibition of miR-140 can significantly reduce the incidence of AD through the activation of PINK1-mediated mitophagy [76]. At the same time, the overexpression of miR-101a can indirectly activate autophagy by regulating mitogen-activated protein kinase (MAPK) [77], suggesting that miRNA-mediated autophagy as an important regulatory role is involved in AD. The timely clearance of Aβ and tau proteins in AD models may be the crucial factors for the alleviation of AD. Relevant studies have also found that the expression level of miR-9-5p is significantly decreased in AD models, and the up-regulation of miR-9-5p can target the ubiquitination factor E4B (UBE4B) and stress-induced phosphoprotein 1 (STIP1) homology and U-box containing protein 1 (STUB1) to activate autophagy and promote the degradation of tau protein, thereby alleviating AD symptoms [78]. In the APP/PS1 mouse model, it has been found that both miR-331-3p and miR-9-5p reveal an obvious decrease at the early stage of AD, and a sequential increase at the late stage of AD, accompanied by abnormal functional changes in autophagy. The suppression of miR-331-3p and miR-9-5p may be associated with targeting autophagy-related proteins SQSTM1/p62 and optineurin (OPTN) to accelerate Aβ clearance and improve cognitive capacity [79]. In the PC12 cell model of AD, the overexpression of let-7a can exacerbate the toxicity of Aβ1-40 to neurons by impaired autophagy through the inhibition of the PI3K/Akt/mTOR signaling pathway [80]. In addition, inhibiting the elevation of miR-17 in AD microglia can activate autophagy, accelerate the clearance of Aβ, and mitigate the progression of AD [81]. Mitochondrial function is also considered to be an important factor in the occurrence and development of AD. In AD models, the elevation of miR-204 can aggravate neuronal ROS production and suppress mitophagy by inhibiting transient receptor potential mucolipin 1 (TRPML1); in contrast, inhibiting miR-204 can rescue this phenomenon [82]. The up-regulation of miR-140 in AD can inhibit the mitophagy-related protein PINK1, and the inhibition of miR-140 can promote the elevation of mitophagy-related protein PINK1, Beclin1, and LC3-II/LC3-I ratio, thereby improving mitochondrial quality [76]. The elevation of miR-351-5p in hippocampal tissues of AD mice can exacerbate mitochondrial fission and mitophagy by targeting mitochondrial Rho GTPase 2 (Miro2), which may be a potential therapeutic target for AD [83]. Moreover, nervous system inflammation is a concomitant state in the formation and development of AD, thereby modulating neuroinflammation as a potential target for AD. The miR-223 can alleviate neuroinflammation by regulating Atg16lL [84], which may be a potential factor for regulating autophagy and inflammation in AD. The function and state of hippocampal neurons also play a crucial role in the progression of AD. Reversing the reduced miR-16-5p in hippocampal tissues of AD mice can improve neurological function and suppress the neurological deficits in AD mice through inhibiting neuronal apoptosis, and exhibiting higher neuronal viability [85]. The elevation of miR-214-3p and miR-124 in hippocampal tissues and cerebrospinal fluid of AD mice can activate autophagy, suppress apoptosis, reduce BACE1 activity, alleviate AD symptoms, and enhance learning and memory capacity [86,87,88]. Moreover, the inhibition of miR-299-5p can significantly promote the activation of autophagy, inhibit apoptosis, and enhance the cognitive capacity of AD mice [89]. The elevated levels of miR-34a in the brain tissues of aging and AD patients can aggravate the occurrence and progression of AD by promoting neuronal apoptosis, reducing synaptic plasticity and function, aggravating Aβ accumulation, and inhibiting autophagy [90]. The overexpression of miR-23b in neurons can reduce neuronal apoptosis, injury, and cognitive impairment by activating Atg12 [91], suggesting its potential significance for the prevention and treatment of AD (Figure 2). Therefore, miRNAs have potential regulatory roles in the clearance of Aβ, tau protein and impaired mitochondria, the reduction of neuroinflammation and neuronal damage, and the suppression of apoptosis, as well as the improvement of neuronal viability by regulating the expression of autophagy-related proteins.

## 6. Potential Strategies for Regulating miRNA-Mediated Autophagy in Alleviating AD

The potential role of miRNAs in regulating autophagy in AD has been gradually reported. However, due to the structural instability and easy degradation, as well as multiple targets of some miRNAs, the specific targeting of miRNAs for application in AD has also become the next issue worthy of exploration. Relevant studies have found that the miRNAs with the regulatory function of autophagy following exercise, drug intake, and other interventions still have great potential for the prevention and treatment of AD in the future, once the specific targets are identified.

### 6.1. Exercise

**Figure 3:**
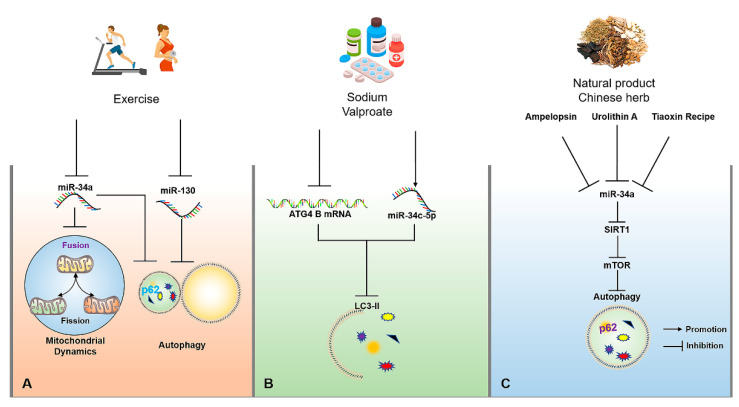
The molecular mechanisms of miRNAs for regulating autophagy in AD upon exercise, drug, natural product and traditional Chinese herb interventions. (**A**) Appropriate exercise can improve mitochondrial dynamics by inhibiting miR-34a and activate autophagy by inhibiting miR-34a and miR-130. (**B**) Sodium valproate can reduce the expression of LC3-II by up-regulating the expression of miR-34c-5p and reducing the stability of ATG4B mRNA. (**C**) Tiaoxinfang, Ampelopsin and urolithin A can delay brain aging through the activation of autophagy by inhibiting miR-34a/SIRT1/mTOR signaling pathway. The → and ┤ in black color represent the promotion and inhibition, respectively.

Exercise has been recognized as an important way to accelerate energy consumption and activate autophagy. Exercise-induced autophagy plays an active role in the prevention and treatment of AD, which may be controlled by the targeted miRNAs to some extent. For example, swimming can significantly inhibit miR-34a-mediated autophagy disorder in hippocampal tissues of aging mice, thereby rescuing abnormal mitochondrial dynamics, and impaired learning and cognitive capacity, as well as achieving the early prevention and treatment of AD [92]. In addition, another study has also found that aged mice subjected to voluntary wheel running reveal the activation of autophagy by down-regulating miR-130 in hippocampal tissues, thereby exhibiting the enhanced learning and memory capacity of aged mice (Figure 3A) [93]. Although relevant studies on regulating miRNA-mediated autophagy upon exercise interventions for AD are limited, miR-34a and miR-130 may be important factors for exercise-induced autophagy, and they also have the potential to be considered as exercise mimics with more critical practical significance for the prevention and treatment of AD in patients with severe AD, or people with movement disorders in the future.

### 6.2. Medicinal Therapy

Medicinal therapy is a meaningful strategy to alleviate or terminate the progression of the disease. Currently, a large number of drugs have been developed based on their targeted miRNAs. For example, donepezil, as an AD treatment drug targeting acetylcholine, has been found in subsequent studies to indirectly up-regulate BDNF in the brain by inhibiting miR-206 [94]. As an α2-adrenoceptor agonist, Dexmedetomidine is often used as a sedative, as shown in rescuing the impaired learning and memory capacity of AD mice through suppressing miR-129-mediated neuronal apoptosis through yes-associated protein 1/Jagged-1 (YAP1/JAG1) axis [95]. As a psychoactive drug, sodium valproate can significantly reduce ATG4B mRNA stability and the expression of LC3-II in the SH-SY5Y cell model of AD through up-regulating the expression of miR-34c-5p, ultimately inhibiting autophagy (Figure 3B) [96]. Therefore, these neurological drugs may be related to miRNA-regulated autophagy in disease control, which may provide a valuable reference point for miRNA-targeted regulation of autophagy to intervene in AD.

### 6.3. Natural Products and Chinese Herbs

Besides exercise and drug interventions, natural products and traditional Chinese herbs also have the potential to regulating miRNA-mediated autophagy for the prevention and treatment of AD. For example, urolithin A, a natural metabolite in pomegranates, fruits, and nuts, can suppress D-galactose (D-gal)-induced brain aging by inhibiting the miR-34a-mediated SIRT1/mTOR signaling pathway to optimize the functional status of autophagy and execute neuroprotection [97]. In addition, Ampelopsin, also known as dihydromyricetin, can delay D-gal-induced brain aging in rats by inhibiting miR-34a and inducing autophagy via the activation of the SIRT1/mTOR signaling pathway [92,98]. Tiaoxinfang is widely used as a prescription for the prevention and treatment of AD in China. It was found that Tiaoxinfang can rescue the impaired learning and cognitive capacity of AD mice by inhibiting miR-34a in hippocampal and cortical tissues (Figure 3C) [99]. Similarly, the regulatory effects of curcumin [100], resveratrol [101], and some Chinese herbs [102] on autophagy have a positive role in the prevention and treatment of AD, but whether the autophagy is mediated by miRNA following the consumption of these natural products needs to be further confirmed. Therefore, these natural products or Chinese herbs with the function for regulating miRNA-mediated autophagy are also important resources that are worthy of in-depth development for targeted intervention in AD.

## 7. Conclusions

As a neurological disease, AD has gained more and more attention with the rapid progress of global aging. A growing number of studies have also documented the regulatory roles of miRNAs in autophagy, suggesting that specific regulating miRNA-mediated autophagy may be a novel and effective strategy for the prevention and treatment of AD. Therefore, this article may provide an important reference point for the early diagnosis, treatment, and rehabilitation of AD by summarizing the potential mechanisms of miRNA-mediated autophagy in AD. It is worth noting that exercise, drug, natural product and Chinese herb interventions can rescue the impaired functional status of autophagy through regulating miRNAs, suggesting their great potential value in the prevention and treatment of AD, as well as the great potential and prospect for exploring exercise mimetics in the future; however, the identification of miRNAs for specifically targeting the signal pathways associated with autophagy is still limited, and targeted regulation of miRNA and binding to specific mRNAs may be important obstacles to future disease interventions.

## Figures and Tables

**Table 1:** The miRNAs involving the regulation of AD in clinical studies.

Model	Tissue	miRNA	Regulatory Roles in AD	References
AD patients	Serum	miR-24-3 ↑	Neuronal apoptosis	[48]
AD patients	Serum	miR-193a-3p ↓	neuronal apoptosis	[49]
AD patients	Serum	miR-4422-5p ↓	Targeting GSAP and BACE1	[50,51]
AD patients	Serum	miR-148a-3p ↓	Targeting ROCK1 to exacerbate Aβ25-35 toxicity to neurons	[52]
AD patients	Serum	miR-222 ↓	Potential biomarker	[53]
AD patients	Serum	miR-223 ↓		[54]
AD patients	Serum	miR-29c-3p ↓		[55]
AD patients	Serum and hippocampus	miR-501-3p ↑		[56]
AD patients	Serum	miR-331-3p ↓	Exacerbating neuroinflammation in Aβ1-40-treated SH-SY5Y cells	[57]
AD patients	Plasma and Cerebrospinal fluid	miR-1273g-3p ↑	Promoting Aβ production and mitochondrial damage	[58]
AD patients	Hippocampus	miR-146a ↑	Exacerbating hyperphosphorylation of tau protein	[60]
AD patients	Hippocampus	miR-146a ↑, miR-181a ↑	Reducing the volume of hippocampus, CA1 and subiculum regions	[61]
AD patients	Olfactory mucosal	miR-206 ↑	Positively correlated with AD patients	[62]
AD patients	Plasma and cerebrospinal fluid	miR-34a-5p ↑	Early potential biomarkers	[59]

Note: the ↑ and ↓ represent the up-regulated and down-regulated microRNAs, respectively.

**Table 2:** miRNAs involving the regulation of AD in animal and cell model studies.

Model	Tissue	miRNA	Regulatory Roles	References
AD cell models	SH-SY5Y and SK-N-SH cells	miR-433 ↓	Targeting JAK2 to inhibit neuronal activity	[63]
AD mice	Hippocampus	miR-613 ↑	Targeting BDNF mRNA to reduce BDNF level	[65]
AD mice (APP/PS1)	Hippocampus, cerebrospinus, and plasma	miR-206 ↑	Reducing BDNF level	[64]
AD mice (5×FAD)	Hippocampus	miR-200c ↓	Targeting 14-3-3γ to increase tau phosphorylation and exacerbate cognitive impairment	[66]
AD mice (APP/PS1) and cell models	Hippocampus/ SH-SY5Y cells	miR-361-3p ↓	Targeting BACE1 to exacerbate Aβ deposition and apoptosis	[71]
AD mice (5×FAD)	Hippocampus	miR-30b ↑	Disrupting hippocampal synapse structure and function	[74]
AD and miR-34c overexpression mice	Hippocampus	miR-34c ↑	Reducing neuronal dendritic spines and synaptic plasticity	[59,67]
AD rats and mice (3×Tg)	Hippocampus	miR-155 ↑	Inducing neuroinflammation and apoptosis	[69,70]
AD mice (SAMP8)	Hippocampus	miR-340 ↓	Targeting BACE1 to exacerbate Aβ deposition and apoptosis	[72]
AD mice (3×Tg)	Hippocampus	miR-128 ↑	Targeting PPARγ to exacerbate Aβ deposition, APP formation and neuroinflammation	[73]
AD mice	Hippocampus	miR-98 ↓	Inhibiting Notch signal pathway, promoting Aβ deposition, exacerbating oxidative stress and mitochondrial dysfunction and apoptosis	[75]
AD rats	Hippocampus	miR-29c-3p ↓	Targeting BACE1 to inhibit Wnt/β-catenin signal pathway	[68]

Note: the ↑ and ↓ represent the up-regulated and down-regulated microRNAs, respectively.

## Data Availability

Not applicable.
